# Optical, Structural, and Charge Transport Properties of Individual Ti_3_C_2_T_*x*_ MXene Flakes via Micro-Ellipsometry and Beyond

**DOI:** 10.1021/acsnano.5c06938

**Published:** 2025-09-30

**Authors:** Ralfy Kenaz, Saptarshi Ghosh, Mailis Lounasvuori, Namrata Sharma, Sergei Remennik, Atzmon Vakahi, Hadar Steinberg, Tristan Petit, Ronen Rapaport, Andreas Furchner

**Affiliations:** † Racah Institute of Physics, 26742 The Hebrew University of Jerusalem, Jerusalem 9190401, Israel; ‡ 28340 Helmholtz-Zentrum Berlin für Materialien und Energie GmbH, Nanoscale Solid−Liquid Interfaces, Schwarzschildstraße 8, 12489 Berlin, Germany; § Center for Nanoscience and Nanotechnology, The Hebrew University of Jerusalem, Jerusalem 9190401, Israel

**Keywords:** Ti_3_C_2_T_*x*_ MXenes, 2D materials, micro-ellipsometry, charge transport, dielectric function, optical constants, structural properties

## Abstract

MXenes have attracted significant attention in recent years due to their remarkable properties for electrochemical and optoelectronic applications. While the physical properties of MXene thin films, consisting of stacked delaminated flakes, have been extensively studied, the intrinsic MXene properties can only be derived from individual flakes. Indeed, flake interconnectivity, intercalated species, and film morphology introduce extrinsic factors that affect charge transport and optical properties. In this work, we quantitatively characterize the intrinsic optical, structural, and transport properties of micrometer-sized Ti_3_C_2_T_*x*_ MXene flakes by employing our non-invasive, advanced spectroscopic micro-ellipsometry (SME) technique in the visible–near-infrared spectral range. SME exploits back-focal-plane imaging in a reflection microscopy geometry to simultaneously capture the spectral and incidence-angle-dependent optical response of individual flakes with up to diffraction-limited lateral resolution. Through a comprehensive multi-flake analysis, encompassing flakes from mono- to 32 layers, we reveal thickness-dependent variations in the complex refractive index and charge transport properties of ultrathin flakes, where resistivity increases as the number of Ti_3_C_2_T_*x*_ layers (NoLs) decreases. Flake thicknesses, non-uniformities, and NoLs, determined via SME with sub-nm precision, closely match nanoscale observations from atomic force microscopy (AFM) and scanning transmission electron microscopy (STEM). Additionally, charge transport properties derived from SME agree with four-probe measurements performed on single-flake devices. Unveiling the intrinsic optical, structural, and charge transport properties of Ti_3_C_2_T_*x*_ MXene single flakes, this study establishes SME as a robust platform for quantitative MXene analyses, enabling precise optical metrology of MXene-based optoelectronic and electrochemical devices.

## Graphical abstract

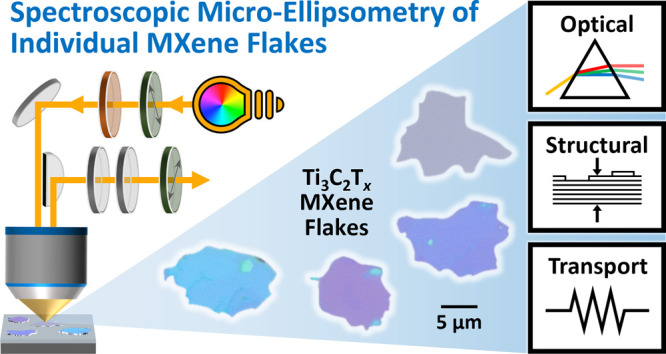

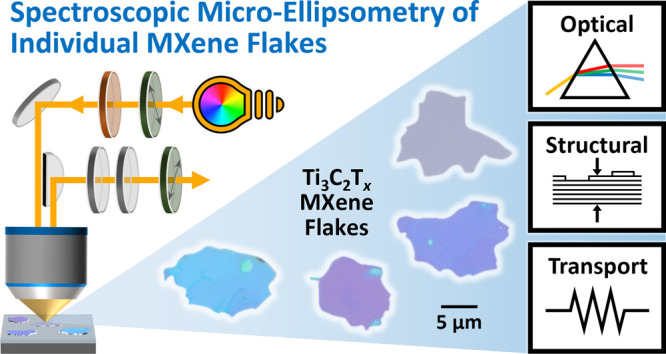

## Introduction

MXenes are emerging as a class of 2D materials with outstanding properties for electrochemical^1,2^ and optoelectronic applications.^3–5^ Their unique combination of high conductivity, environmental stability, and tunable surface chemistry offers a versatile platform for designing novel photodetectors,^6,7^ sensors,^8^ artificial synapses,^9^ and energy-storage devices.^1,2,10^

To date, most MXene applications rely on thin films prepared by the stacking of delaminated MXene flakes. In such systems, MXene composition,^11,12^ number of layers,^13^ morphology,^14–16^ and intercalated species^3,17^ were found to influence the optical film properties. Charge transport was also observed to be significantly affected by flake-to-flake connectivity and intercalated water or ions.^18–21^ Fundamental studies on individual MXene flakes are therefore essential to distinguish the intrinsic electronic and optical properties from extrinsic MXene properties.

For few-layered Ti_3_C_2_T_*x*_ MXene flakes, the number of layers was found to affect electrical conductivity,^22–24^ oxidation stability,^25^ and plasmonic properties.^26,27^ However, establishing structure–function relationships at the single-flake level remains challenging, as different techniques are typically required to assess structural, chemical, optical, and functional properties. For structural properties, atomic force microscopy (AFM)^23,28^ and (scanning) transmission electron microscopy (STEM) are commonly employed.^29,30^ Chemical information on individual flakes can be obtained using tip-enhanced Raman spectroscopy (TERS),^31^ electron energy loss spectroscopy (EELS),^26,32^ or X-ray microscopy.^33,34^ Optical properties may vary from flake to flake, which requires hyperspectral imaging of single flakes.^35^ Finally, charge transport properties are usually derived through device fabrication^22,23^ and direct electrical measurements.

Ellipsometry^36,37^ is widely used on thin films to probe the optical properties and derive correlations between the structural and functional properties. As a sensitive, non-destructive optical technique with sub-nanometer thickness precision, ellipsometry measures the change in polarization state of light upon interaction with a sample, thus probing its physical properties. Ellipsometry has previously been deployed to investigate MXene films for their optical properties.^21,38–44^ However, due to the inherent limitations of conventional ellipsometry in measuring lateral areas smaller than 50 μm, individual MXene flakes with micron-scale lateral dimensions could not yet be investigated ellipsometrically.

We recently developed an advanced technique for performing spectroscopic micro-ellipsometry (SME) via back-focal-plane (Fourier plane) imaging in a reflection microscopy geometry.^45^ This method provides diffraction-limited lateral resolution (≤5 μm spot size) and the simultaneous acquisition of broadband spectral ellipsometry data at multiple incidence angles in under a minute. SME thus breaks the barriers of conventional ellipsometry regarding lateral resolution and required measurement times, and is therefore tailor-made for studying microstructures such as individual MXene flakes. Its sensitivity to even single atomic layers has been previously demonstrated on exfoliated 2D materials with micron-scale lateral dimensions.^45,46^

In this work, we employ SME in the visible–near-infrared (Vis–NIR) spectral range to determine the optical, structural, and charge transport properties of individual Ti_3_C_2_T_*x*_ MXene flakes (Figure 1). Performing a comprehensive multi-sample analysis of 24 flakes, ranging from mono- to few-layer configurations with up to 32 stacked Ti_3_C_2_T_*x*_ layers, we quantify the flake-thickness-dependent optical constants and dielectric function (complex permittivity). Analyzing the variations in the intrinsic optical properties, we extract the flakes’ charge transport characteristics (resistivity, sheet resistance, scattering time), and validate the observed resistivity behavior via direct electrical four-probe measurements on MXene single-flake devices. Lastly, we gain deep insights into the structural flake properties. The ellipsometrically determined flake thicknesses, numbers of Ti_3_C_2_T_*x*_ layers (NoL) within the flakes, and flake non-uniformities are corroborated by topography measurements with AFM, and nanoscale thickness and NoL measurements with STEM. This study significantly advances the understanding of individual 2D MXene flakes, paving the way for their effective integration into next-generation optoelectronic devices and broadening perspectives for probing charge storage mechanisms in single flakes.

**Figure 1:**
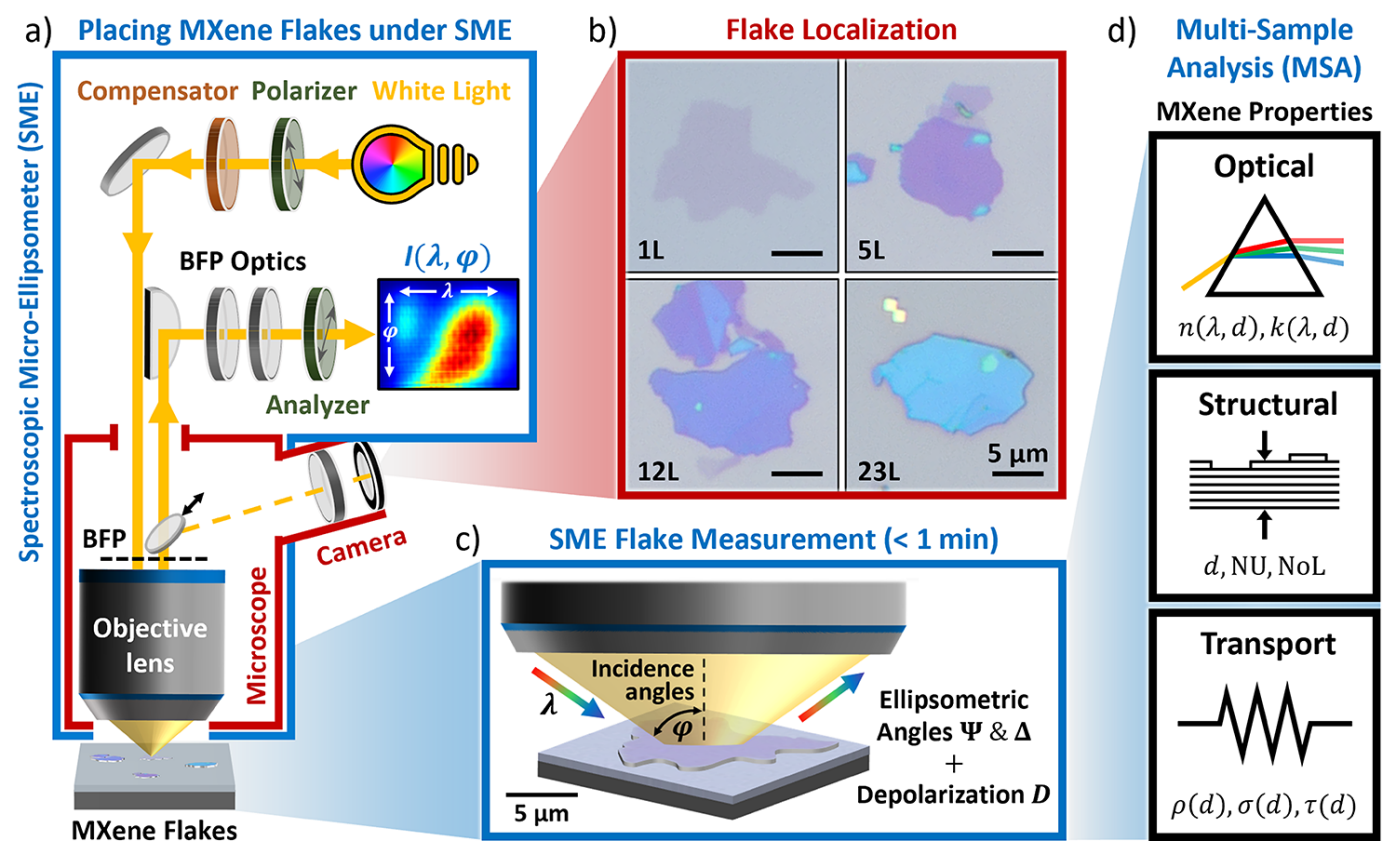
Spectroscopic micro-ellipsometry (SME) workflow for the characterization of individual MXene flakes on SiO_2_/Si substrate.(a) Schematic of the SME, detailed in our previous work.^45^ (b) MXene flakes with different optical contrast (L is the number of Ti_3_C_2_T_*x*_ layers) are located on the substrate using the camera of the SME microscope. (c) Switching from sample-plane imaging microscopy mode [*I*(*x, y*)] to back-focal-plane (BFP) imaging ellipsometry mode [*I*(λ, φ)], SME measurements with a spot size of 5 μm are performed on the individual flakes in under 1 min per flake. (d) The resulting spectral (λ), multi-incidence-angle (φ) ellipsometric parameters (amplitude Ψ, phase Δ, and depolarization *D*) are used in a multi-sample analysis to extract the optical, structural, and charge transport properties of the MXene flakes (see text).

## Results and Discussion

### Ellipsometric Single-Flake Characterization

Mono- to few-layered Ti_3_C_2_T_*x*_ MXene was synthesized via wet-chemical etching of a Ti_3_AlC_2_ MAX phase in HF/HCl, followed by delamination in LiCl (details in Materials and Methods). MXene was dropcast onto a silicon wafer with a 289 nm thick thermal oxide (SiO_2_) layer for enhanced optical contrast.^47^ The concentration of the dropcast dispersion was optimized to obtain well-separated individual flakes. MXene flakes of different thicknesses were screened for SME analysis using the white-light microscopy function of the SME instrument (Figure 1b). Five monolayer (1L) flakes, three bilayer (2L) flakes, two trilayer (3L) flakes, and two four-layer (4L) flakes were identified and measured with SME (Figure 1c), allowing us to test the reproducibility, consistency, and statistical robustness of the ellipsometric data. Additionally, we measured twelve few-layer flakes, ranging from five (5L) to 32 layers (32L). These NoL values are a key outcome of SME, which we discuss later on.

Ellipsometry measures the ratio of *p*- to *s*-polarized complex reflection coefficients, *r*_p_/*r*_s_ = tan Ψ e^*i*Δ^, expressed by the ellipsometric angles Ψ (amplitude) and Δ (phase), as well as the depolarization *D*, which is related to sample non-idealities.^36,37,48^ Figure 2a shows single-incidence-angle Ψ spectra of all 24 identified Ti_3_C_2_T_*x*_ flakes (Δ and *D* spectra in Figure S1), as well as corresponding flake thicknesses derived from the ellipsometric analysis. The Ψ measurements are visibly sensitive to thickness and, consequently, to the number of Ti_3_C_2_T_*x*_ layers within each flake. The spectra are dominated by the film interference signature related to the thick SiO_2_ layer beneath the MXene flakes, which serves as a sensitive indicator of flake thickness. Modulated by the optical and structural properties of the flakes, this signature redshifts with increasing flake thickness from 503 nm for monolayer flakes to 568 nm for the 32L flake. The oxide interference also allows one to conveniently distinguish between thinner and thicker flakes. At 45° incidence angle, thin flakes between one and eight layers give rise to an upward-pointing signature in Ψ, while flakes with more than eight layers lead to a downward-pointing signature.

**Figure 2:**
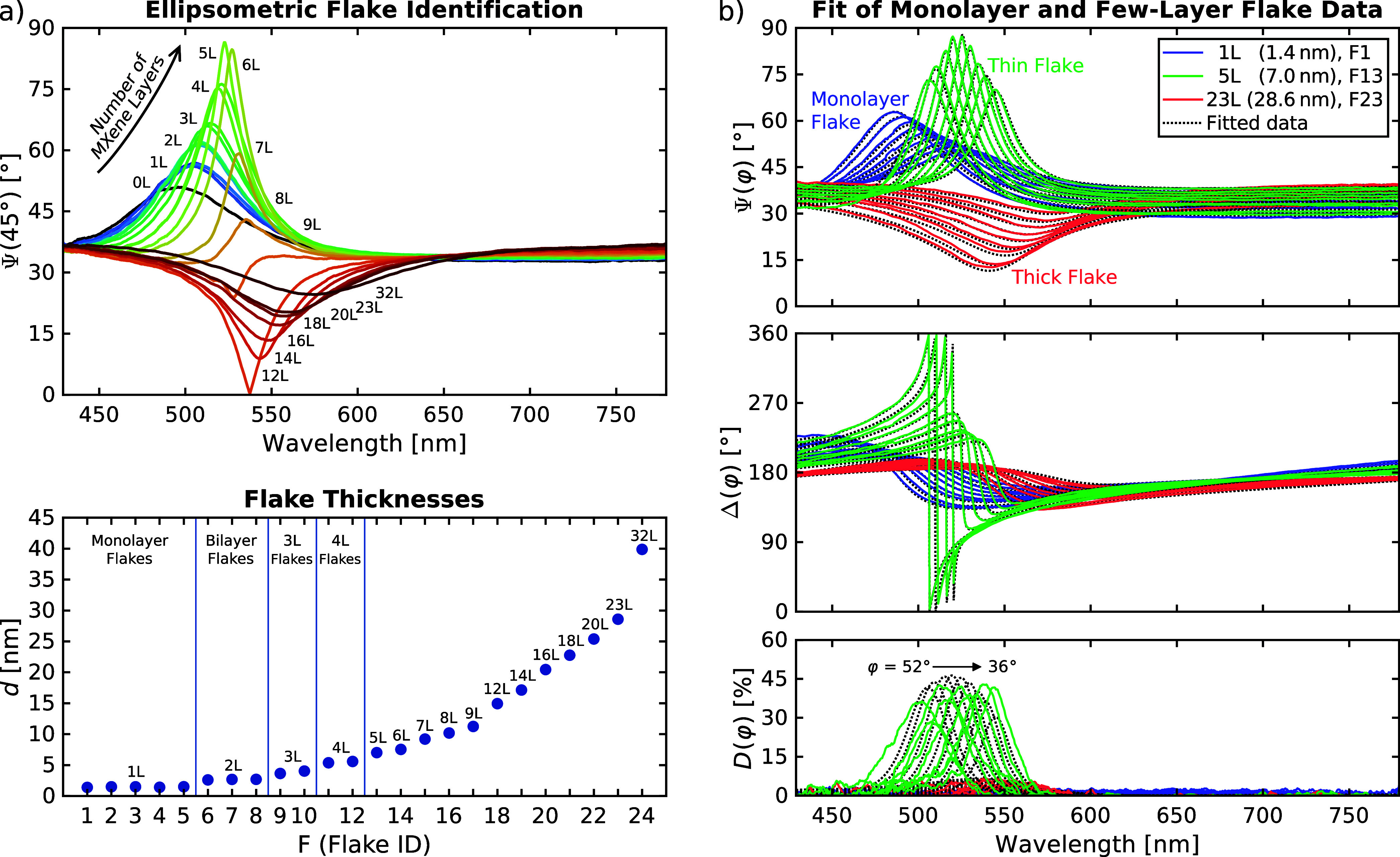
(a) Flake survey of all 24 investigated Ti_3_C_2_T_*x*_ flakes, showing ellipsometric Ψ spectra at 45° incidence angle (with indicated NoL for each flake), and corresponding flake thicknesses *d* determined from SME. (b) Measured and fitted ellipsometric amplitude (Ψ), phase (Δ), and depolarization (*D*) spectra of a monolayer, a thin, and a thick Ti_3_C_2_T_*x*_ flake for incidence angles φ between 52° and 36°.

The measurements of the five different monolayer flakes and the three bilayer flakes are reproducible and self-consistent, demonstrating the homogeneity of the investigated thin flakes with one or two layers. All flakes in our study with three or more layers turn out to be non-uniform, as is already evident from the two slightly differing 3L and 4L Ψ spectra. Correspondingly, the homogeneous 1L and 2L flakes exhibit optically determined flake thicknesses of *d*_mono_ = 1.40–1.51 nm and *d*_bi_ = 2.61–2.70 nm, whereas slight thickness differences are observed in the two 3L and in the two 4L flakes. Naturally, flake thickness increases with NoL, reaching up to 39.9 nm for the 32L flake. The SME-derived flake thicknesses are in close agreement with AFM and STEM results (Figure S2).

We now focus on the ellipsometric analysis to extract the optical and material properties of individual MXene flakes. Figure 2b shows measured and fitted multi-angle Ψ, Δ, and *D* spectra of a monolayer (1L), a five-layer (5L) thin, and a 23-layer (23L) thick flake. Each flake exhibits a characteristic ellipsometric fingerprint in Ψ and Δ, related to both flake thickness and optical properties. Furthermore, flake non-uniformity gives rise to depolarization *D*, an important observable uniquely accessible through ellipsometry. *D* values greater than zero indicate that a MXene flake consists of different numbers of Ti_3_C_2_T_*x*_ layers within the SME measurement spot.

Excellent agreement between experimental and theoretical ellipsometric data is achieved by incorporating into the optical model^36,37^ the optical, transport, and structural properties of the MXene flakes. Analogous to the nature of the MXene samples in this study, the optical model consists of Si substrate, 289 nm SiO_2_, and MXene on top. The MXene is parameterized by its thickness, its thickness non-uniformity, and its wavelength-dependent dielectric function ϵ­(λ). The UV–Vis–NIR spectral range of ϵ­(λ) is governed by at least seven resonances related to various interband transitions, with additional intraband transitions giving rise to the metallic Drude conductivity in the mid-IR.^42–44^ From the Vis–NIR region accessed with our micro-ellipsometer, we can extract ϵ­(λ) as the sum of a Drude oscillator and two harmonic oscillators, ϵ­(λ) = 1 + ϵ_Drude_(λ) + ϵ_Harm_^NIR^(λ) + ϵ_Harm_^UV^(λ). The Drude oscillator accounts for the free-charge-carrier properties (resistivity ρ, mean scattering time τ) of the conductive MXene flakes. The NIR oscillator, with a fitted position of 820 nm (1.5 eV), is widely discussed in the literature as being of plasmonic and/or interband origin, likely associated with Ti_3_C_2_O_2_ termination.^11,26,39,43,44,49^ Pronounced UV absorptions are expected below 450 nm (2.7 eV).^42,44^ The corresponding UV oscillator, fitted to 240 nm (5.1 eV), represents the spectral overlap associated with different interband transitions from mixed Ti_3_C_2_T_*x*_ terminations (T = O, OH, Cl, F) that cause absorption outside and at the edge of the measured spectral range below 429 nm (2.9 eV). No additional UV or IR poles were used in the dielectric function, as these would mask the correct values of the Drude parameters.

Furthermore, we aimed at pinpointing those MXene properties that differ from flake to flake by employing a multi-sample analysis (MSA), in which we initially allowed all samples to share the same common parameters, except for the flake thickness. We then identified two additional flake-specific properties, namely, the thickness non-uniformity and the resistivity ρ, both of which influence the quantification of the MXene optical response (details in Materials and Methods).

### Optical Properties of MXene Flakes

Figure 3 shows the dielectric function (complex permittivity) ϵ = ϵ_1_ + *iϵ*_2_ = (*n* + *ik*)^2^ and optical constants *n* (refractive index) and *k* (absorptive index) resulting from the ellipsometric MSA, where ϵ_1_ = *n* ^2^ – *k* ^2^ and ϵ_2_ = 2*nk*. These properties exhibit small variations with flake thickness (or number of layers), mainly for the 1L and 2L flakes in this study. ϵ_1_ and ϵ_2_ (*n* and *k*) are dominated by the Ti_3_C_2_O_2_-related NIR oscillator at 820 nm (1.5 eV), the spectral position of which is in agreement with previous observations from macroscopic multi-flake films.^11,21,39,41–44^ Overall, the dependence of ϵ on thickness is small and primarily affects the real part ϵ_1_ and the optical constants. Note that this dual influence on *n* and *k* impacts the ellipsometric baselines of both Ψ and Δ, rendering SME highly sensitive toward the flake properties.

**Figure 3:**
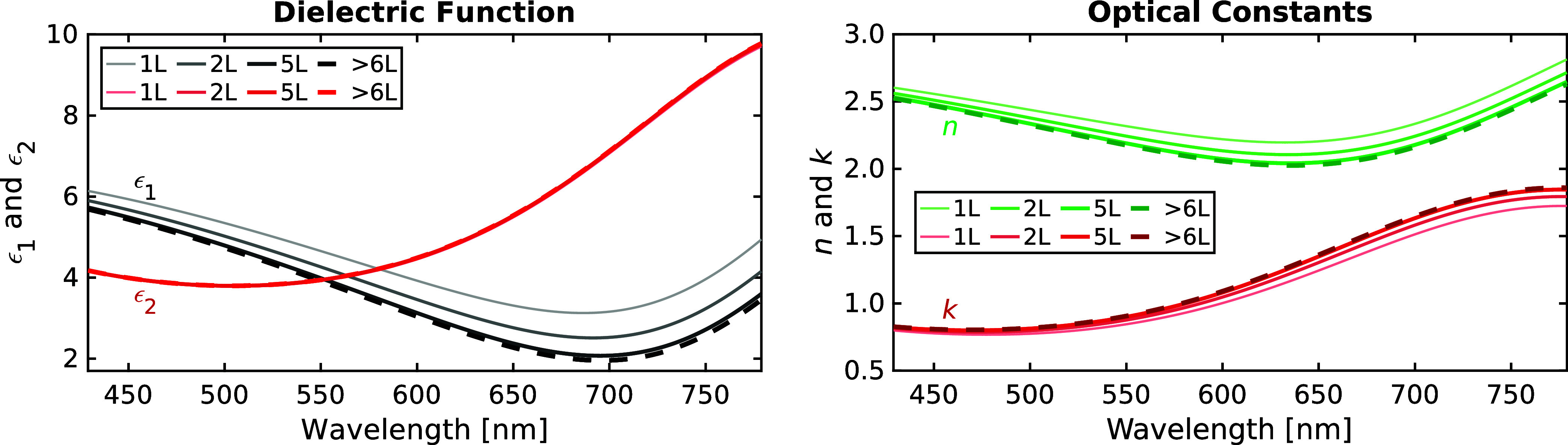
Dielectric function (real part ϵ_1_, imaginary part ϵ_2_) and optical constants (refractive index *n*, absorptive index *k*) for flakes with different numbers of layers. Having included five 1L and three 2L flakes in the MSA renders the results statistically robust. Plots with eV scales are given in Figure S3.

The optical characterization of individual Ti_3_C_2_T_*x*_ MXene flakes highlights that their intrinsic optical properties in the Vis–NIR spectral range are comparable to those of thick films^21,38–44^ (e. g., from spin- or spraycoating), which are composed of multiple stacked mono- or few-layered flakes. However, the variations among the reported optical properties of macroscopic films are much larger than the relatively small thickness-dependent variations we observe between individual Ti_3_C_2_T_*x*_ flakes. This suggests some influence of the films’ structural properties on their optical properties, where the dependence of ϵ on thickness or structure may be related to depth-dependent flake-to-flake interconnectivity, flake-stacking, porosity, and/or oxidation effects rather than to the intrinsic properties of the flakes themselves.

The dielectric function (or optical constants) can be used to calculate transmission and absorbance spectra of all flakes (Figure S4). Furthermore, it provides quantitative information on the flakes’ transport properties through the Drude parameters ρ and τ.

### Transport Properties of MXene Flakes

The transport properties of Ti_3_C_2_T_*x*_ MXene flakes, extracted from the ellipsometric MSA, are shown in Figure 4a. The resistivity of individual thin Ti_3_C_2_T_*x*_ flakes is found to depend on their thickness. Among the studied flakes, we observe the strongest dependence for flakes with one or two layers, but there is a general trend for flakes thinner than 8 nm (6L), as suggested by the variations in the optical constants. Starting with the highest average resistivity value of ρ_mono_ = (1.56 ± 0.18) × 10^–4^ Ω cm for monolayer flakes, the resistivity decreases with increasing flake thickness and reaches a constant average value of ρ_thick_ = (0.79 ± 0.12) × 10^–4^ Ω cm for flakes with more than six layers (thicker than 8 nm). The corresponding conductivities (σ = 1/ρ) are σ_mono_ = (6410 ± 740) S cm^–1^ and σ_thick_ = (12700 ± 1900) S cm^–1^. Sheet resistance (the ratio of resistivity to thickness) decreases with increasing thickness, with the highest value of *R*_mono_ = (1.09 ± 0.12) kΩ/□ found for monolayer flakes. The mean scattering time of τ_flake_ = (8 ± 3) fs is found to be independent of thickness.

Because the Drude and NIR oscillator in Ti_3_C_2_T_*x*_’s dielectric function are situated at the edge of the measured spectral range, we explored alternative models (see Materials and Methods) to assess the influence of the NIR resonance. We found that the transport properties derived from the presented model are robust. However, since single-flake ellipsometry studies are uncharted territory, we corroborated the SME results with a complementary non-optical method. Specifically, we made devices of several Ti_3_C_2_T_*x*_ flakes using four-probe contact geometries (Figure 4b). Electrical resistivity measurements, performed on five mono- to few-layer flakes, are in line with the SME findings (Figures 4b and S5), demonstrating constant resistivity for thick flakes, increased resistivity for monolayer flakes, and a 1/*d*-related decrease in sheet resistance with flake thickness.

Our non-invasive optical measurement of ρ_mono_ also agrees with values reported from other groups that carried out direct electrical measurements on similar Ti_3_C_2_T_*x*_ flakes [1.6 × 10^–4^ Ω cm, (2.31 ± 0.57) × 10^–4^ Ω cm, (1.14 ± 0.21) × 10^–4^ Ω cm].^22–24^ Although the literature focuses mainly on monolayer flakes, it does also indicate a possible decrease in flake resistivity with thickness.^22,24,50^ Sheet resistance has been measured on mosaic self-assembled Ti_3_C_2_T_*x*_ films, yielding values of approximately *R*_mosaic_ = 10 kΩ/□, 5 kΩ/□, and 4 kΩ/□ for mono-, bi-, and trilayer films, respectively.^51^ Our values for mono-, bi-, and trilayer flakes show the same decreasing trend but are an order of magnitude smaller, likely because mosaic films are significantly affected by Ohmic losses due to limited connectivity between flakes. This highlights that Ti_3_C_2_T_*x*_-based MXenes could be particularly promising for single-flake optoelectronic devices.

Our optically derived values for resistivity, scattering time, and sheet resistance from SME are in close agreement with those previously determined for spraycoated Ti_3_C_2_T_*x*_ films.^44^ In these films, the transport properties could only be described in terms of a gradient in Drude resistivity and scattering time with film depth. For free charge carriers closest to the substrate, it was found that τ_spray_ = 8.1 fs, which is similar to the value determined in this work. This suggests that the properties of individual Ti_3_C_2_T_*x*_ MXene flakes we studied here resemble those of a closed Ti_3_C_2_T_*x*_ layer of interconnected flakes within the film at that depth. Interestingly, the resistivity changed from ρ_spray_^bottom^ = 0.47 × 10^–4^ Ω cm near the substrate to ρ_spray_^top^ = 0.78 × 10^–4^ Ω cm at the MXene/air interface. The latter value coincides with the resistivity observed here for thicker flakes.

The increase in resistivity in thinner flakes is particularly interesting. While local oxidation due to ambient moisture and water trapped in wrinkles, leading to the formation of TiO_2_ nanoparticles, is a possibility,^21,52^ it is more likely to occur in thicker flakes. In fact, the oxidation stability of monolayer flake was found to be enhanced due to the absence of trapped water.^21^ On the other hand, the top surface of a flake is exposed to air, and molecular adsorbates such as water may lead to p-type doping, inducing a decrease in conductivity.^23^ We therefore suspect the change in resistivity with flake thickness to be related to changes in the surface-to-bulk ratio, corresponding to the ratio of air-exposed surface terminations to interlayer-exposed terminations. In other words, as the number of MXene layers increases, the ratio of surface layer doped by molecular adsorbates to interlayer T_*x*_ groups decreases, becoming negligible for flakes thicker than approximately six layers.

The dielectric function probed with SME could also be influenced by water or optical anisotropy in the in-plane (basal plane) and out-of-plane conductivities.^38,53^ While water likely has little effect on the MXene optical properties in the Vis spectral range (*k*_water_ = 0, *n*_water_ = 1.33 ≈ const.), mid-IR ellipsometry could be helpful for studying the impact of water on the optical and transport properties by directly probing the vibrational signatures of interlayer OH groups.^44^ Ellipsometry extended into the mid-IR would probably be required to probe in detail potential anisotropy effects, and to consolidate the changes in the optical properties and flake conductivity with thickness by reducing the uncertainty in the Drude parameters (particularly in the scattering time τ).

**Figure 4:**
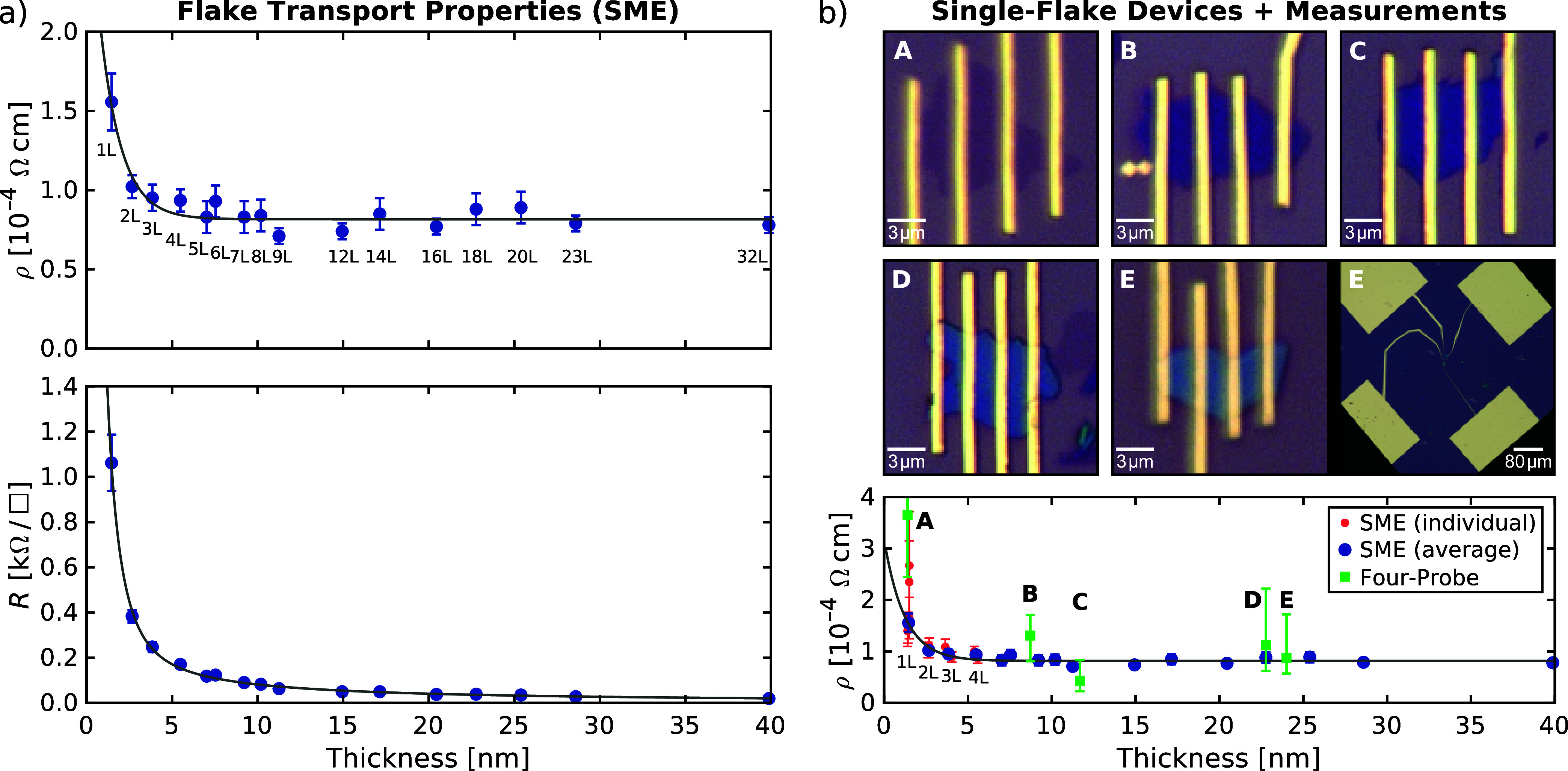
(a) Flake transport properties (resistivity ρ and sheet resistance *R*) obtained from the ellipsometric MSA in dependence of flake thickness and number of Ti_3_C_2_T_*x*_ layers. (b) Optical images of mono- (A) and few-layer (B–E) single-flake Ti_3_C_2_T_*x*_ devices, and four-probe resistivity measurements compared with SME. A wider field of view shows how the four gold contact pads connect to flake E. SME data of the 1L, 2L, 3L, and 4L flakes are shown both as averages and individually for each flake.

### Structural Flake Properties

The multi-sample ellipsometry approach provides detailed information on flake thickness (*d*), number of Ti_3_C_2_T_*x*_ layers (NoL), and flake non-uniformity (NU). Here, we investigate how these properties, determined non-invasively with SME, compare with data from complementary nanoscale methods that are relatively more invasive or even destructive. We used AFM to measure the flake topography within the region probed by SME (Figure S6). STEM cross-section imaging of the flakes then enabled us to directly count the number of MXene layers and study flake non-uniformity at both the nano- and microscale. The results are shown in Figures 5 and S7.

**Figure 5:**
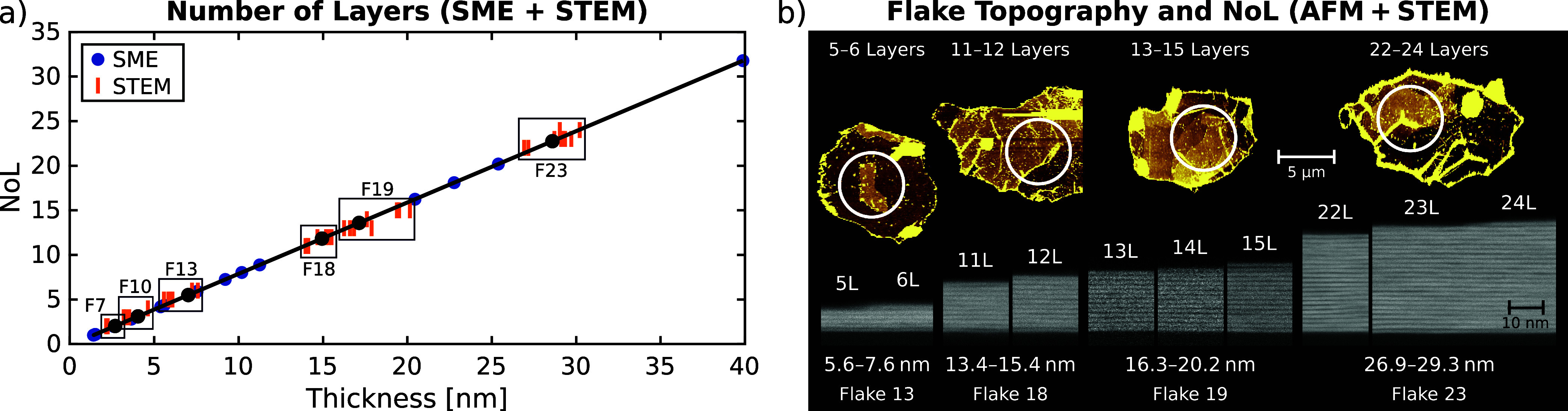
Structural flake properties: (a) Number of Ti_3_C_2_T_*x*_ layers (NoL) of MXene flakes derived from ellipsometry compared with direct layer counts from STEM HAADF images at various points along the SME measurement spots on the flakes. (b) Flake topography (AFM, colors adjusted per flake for optimized NoL contrast), and nanoscale layer thicknesses and NoL (STEM) from selected flakes (circles represent SME spots). STEM of flakes 13 and 23 shows the transitions from 5L to 6L and from 23L to 24L regions, respectively.

With a broad range of SME-derived flake thicknesses from a collection of 24 flakes, we can confidently determine the average NoL for each flake (Figure 5a). Extrapolating from the thicknesses of the uniform mono- and bilayer flakes, we find that each additional MXene layer adds 1.25 nm to the base monolayer thickness of 1.40 nm. The thickest flake (39.9 nm) in our study consists (on average) of 31.8 ± 0.4 stacked MXene layers. STEM-derived thickness and NoL values, taken along several points of the cut flakes, scatter around the SME values, validating the linear dependence of NoL on thickness. Both NoL and flake thicknesses observed with STEM (Figure 5b) show excellent agreement with the ellipsometry results, confirming SME as a reliable non-invasive method for nanoscale thickness quantification. Thus, micro-ellipsometry effectively bridges lateral microscale measurements of individual MXene flakes with precise thickness characterization at the nanoscale.

Note that individual flakes can exhibit variations in the NoL within a single flake, as well as in the STEM-derived thicknesses at a fixed NoL (Figure 5). These variations are quantified by the thickness non-uniformity (NU), which is another important structural parameter of MXene flakes accessible through ellipsometry. We are able to quantify NU accurately because of the contrast-enhancing thick SiO_2_ layer and the broad range of incidence angles simultaneously captured in a single SME measurement. This dual sensitivity enables the detection of even small non-uniformities.

Flake non-uniformities determined from SME, in terms of both thickness (in nm) and number of layers (in NoL), are presented in Figure 6a. Mono- and bilayer flakes turn out to be uniform (NU_*d*_ = 0 nm), as expected for thin and highly ordered 2D flakes. For thicker flakes, non-zero values up to NU_*d*_ = 7 nm (NU_NoL_ = 5 NoL) are observed. This is likely due to increasing disorder and defects within and between the stacked MXene layers.

**Figure 6:**
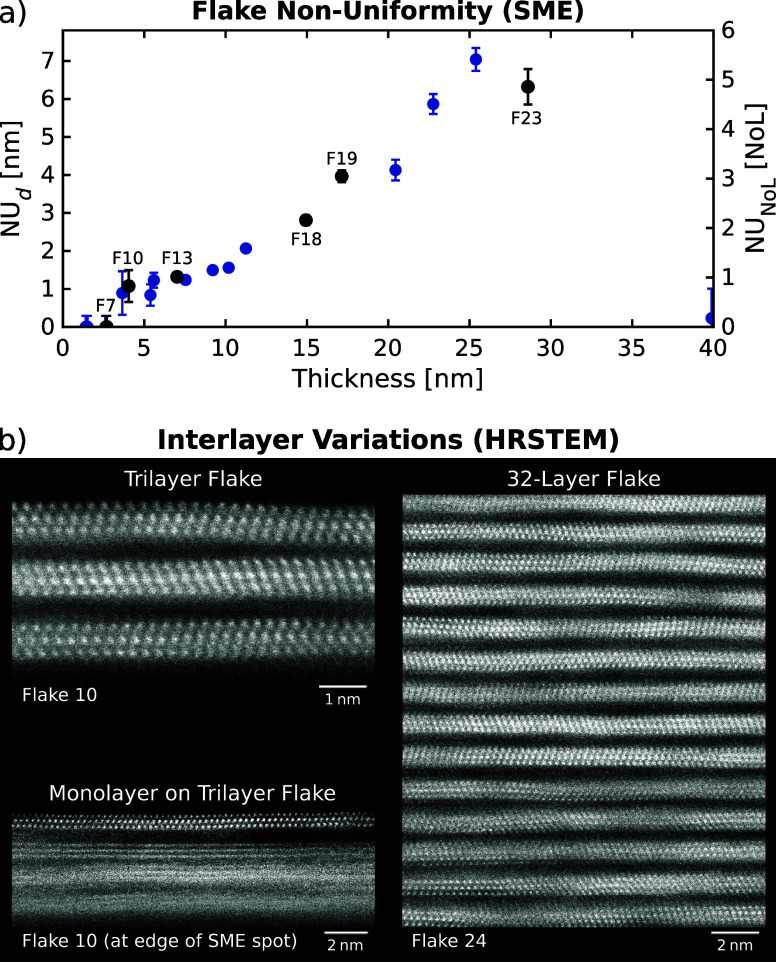
(a) Flake non-uniformity from SME corresponding to the variations in thickness (nm) or NoL of the flakes within the SME spots. (b) Non-uniformity in the interlayer spacing from HRSTEM images of a trilayer flake and part of a 32-layer flake in {1120} orientation of Ti_3_C_2_T_*x*_ ({11̅00} orientation in Figure S8), and a monolayer on a trilayer flake with orientation and interlayer mismatches. Note the characteristic zigzag pattern of Ti atoms (as expected from a *P*6_3_/*mmc* space-group material).

Results for flake thickness, non-uniformity, NoL, and topography from SME, STEM, and AFM are remarkably consistent. For instance, STEM reveals that flake F13 consists of five layers (5L) with a thickness of ≈5.8 nm. However, AFM topography shows that approximately 40% of the central region within the SME spot is covered by an additional layer, increasing the local thickness to ≈7.4 nm. Ellipsometry yields values of *d* = 7.0 nm, NoL = 5.50 ± 0.06, and NU_NoL_ = (1.0 ± 0.1) NoL, consistent with the presence of a partial 6L region. Similarly, flakes F18 and F19 contain, respectively, 11–12 and 13–15 layers, as determined by STEM, in accordance with SME, which gives NoL = 11.8 ± 0.1 and 13.6 ± 0.2, with layer non-uniformities of NU_NoL_ = (2.1 ± 0.1) NoL and (3.0 ± 0.1) NoL.

Lastly, we discuss the significant variances in the STEM-derived thicknesses that occur both at the nanoscale within individual STEM images and at the microscale between images of different flake regions (Figure S7). For instance, the 5L, 13L, and 23L regions of flake F13, F19, and F23 have thicknesses of 5.5–6.0 nm, 16.2–17.9 nm, and 28.7–29.7 nm, respectively. These variances can be larger than the thickness of a monolayer. High-resolution STEM images (Figure 6b) of a trilayer flake and a 32-layer thick flake reveal nanoscale fluctuations in interlayer spacing possibly responsible for these variations. The variability in the distance between layers is likely related to a non-uniform distribution of intercalants (ions) and mixed MXene surface terminations (O, OH, Cl, F).

Furthermore, in HRSTEM, we do not see significant orientation mismatches between stacked layers in individual flakes. This is expected, as the flakes were synthesized from the same initial MAX phase particles and had not been fully delaminated. However, in free-standing films formed by restacking fully delaminated monolayer flakes, orientation mismatches may lead to even greater interlayer-spacing non-uniformities than those observed in our flakes. An example of orientation and interlayer mismatches is shown in Figure 6b, where a trilayer flake is partially covered by an additional monolayer flake. The individual Ti atoms of the monolayer are clearly visible (close to {112̅0} orientation), whereas they are indistinct in the trilayer due to orientation differences. Additionally, the interlayer spacing between mono- and trilayer also differs from the spacings within the trilayer itself.

In summary, SME, AFM, and STEM consistently demonstrate that partially delaminated flakes can have non-uniform thicknesses and numbers of layers. However, these structural variations do not seem to impact the intrinsic optical and transport properties of the flakes. This finding is critical for future applications of MXene flakes as constituents of photonic and electronic devices.

## Conclusions and Outlook

We have analyzed fundamental optical and transport properties of individual Ti_3_C_2_T_*x*_ MXene flakes using spectroscopic micro-ellipsometry (SME), an advanced, non-destructive, high-throughput polarization-dependent optical technique. SME provides sub-5 μm lateral resolution in the Vis–NIR spectral range, with spectrally and angularly resolved ellipsometric data acquired in under 1 min, enabling the effective probing of individual flakes. A comprehensive analysis of 24 flakes, ranging from mono- to 32 layers, allowed us to determine the intrinsic dielectric functions of Ti_3_C_2_T_*x*_ MXene. These optical properties also contain information on charge transport, revealing a pronounced thickness dependence in resistivity for ultrathin flakes, which aligns well with direct electrical four-probe measurements. Structural parameters such as thickness, inhomogeneity, and number of Ti_3_C_2_T_*x*_ layers extracted from SME are in close agreement with AFM and STEM measurements. This work establishes SME as a powerful tool for investigating the intrinsic physical properties of individual MXene flakes, opening additional avenues for optical metrology of MXene-based optoelectronic devices. Beyond that, leveraging both the sensitivity of the MXene optical properties to redox processes^17^ and the non-invasive nature of SME, we envision that SME is ideally suited for operando imaging of electrochemical reactions and intercalation processes^54,55^ in individual MXene flakes.

## Materials and Methods

### Chemicals

Ti_3_AlC_2_ MAX phase (provided by RWTH Aachen, Germany), hydrochloric acid (HCl, ROTH, 37%), hydrofluoric acid (HF, ACS reagent, 48%), and lithium chloride (LiCl, ACROS organic, 99%, extra pure).

### MXene Synthesis

Ti_3_C_2_T_*x*_ MXene was prepared by following an optimized procedure by Mathis et al.^56^ Briefly, the Al layer was etched from the Ti_3_AlC_2_ MAX phase using a mixture of HF, HCl and deionized water in the ratio 1:6:3. 1 g of MAX phase was stirred at 400 rpm in 25 mL of acid mixture for 24 h at 35 °C. The obtained etchant mixture was washed multiple times in distilled water until the pH reached between 5–7. For delamination, the washed multilayer MXene was immersed in 50 mL of 0.5 M LiCl solution and stirred for 18 h at room temperature. The resulting Li^+^-intercalated sheets were then washed twice in distilled water (150 mL each time) by centrifuging at 3500 rpm for 10 min to remove excess Li ions. After a second wash, the supernatant was thick and collected as delaminated mono- to few-layered Ti_3_C_2_T_*x*_ MXene. To increase the stability and shelf life of the MXene, the supernatant was concentrated to 8 mg/mL at 8500 rpm.

### Preparation of MXene Flakes

Silicon (1 × 1 cm^2^) with a 289 nm thick thermal oxide layer (SiO_2_) was used as substrate. A 2D grid of 50 μm spaced gold markers (50 nm Au with 5 nm Ti for better adhesion), deposited after electron-beam lithography (ELS-G100, Elionix) via electron-beam evaporation (Vacuum System Technologies), served the dual purpose of easy MXene flake (re)­localization, and nanoscale accurate deposition of contacts for direct electrical four-probe measurements.^57^ Before MXene deposition, the substrate surface was cleaned by sonication in acetone and isopropanol for 10 min each, followed by UV plasma cleaning for 20 min. 20 μL of diluted Ti_3_C_2_T_*x*_ MXene (0.01 mg/mL) was dropcast onto the substrate, resulting in sparsely deposited individual MXene flakes.

### Spectroscopic Micro-Ellipsometry (SME)

In a single measurement of under a minute, our spectroscopic micro-ellipsometer (SME) captures ellipsometric Ψ, Δ, and *D* data simultaneously at multiple wavelengths and multiple incidence angles, with a diffraction-limited measurement spot size (set to around 5 μm in this study).^45^ This capability is enabled by imaging the back focal plane of the SME’s high-numerical-aperture objective lens (NA = 0.9). With the current optical components, the SME acquires more than 700 wavelength points between 429 nm and 779 nm at over 100 incidence angles between 10° and 63°. To optimize the signal-to-noise ratio and therefore improve the fit quality, we selected 34 incidence angles between 34° and 52° for further analysis. The SME’s high ellipsometric accuracy and performance have been previously demonstrated, along with its high sensitivity in measuring atomically thin 2D materials^45,46^ and its ability to probe the optical properties of microscopic nanoparticle lattices.^58^

Our micro-ellipsometer measures the four Stokes parameters^37,48^ that describe the polarization state of light after interaction with sample and ellipsometer optics. After a calibration procedure of incidence angles and instrumental polarization of the SME system^45^ (done once before the MXene measurements), we obtain the ellipsometric quantities ⟨*N*⟩, ⟨*C*⟩, and ⟨*S*⟩ of the measured flakes, from which we derive the ellipsometric angles Ψ (amplitude) and Δ (phase) according to^48^

1 $$\begin{array}{l} \left\langle N\right\rangle =P\;\cos\;2\Psi ,\\ \left\langle C\right\rangle =P\;\sin\;2\Psi \;\cos\;\Delta ,\\ \left\langle S\right\rangle =P\;\sin\;2\Psi \;\sin\;\Delta ,\\ P^{2}=\langle N\rangle^{2}+\langle C\rangle^{2}+\langle S\rangle^{2},\\ D=1-P^{2}, \end{array}$$

with the polarization degree *P* and the depolarization *D*. The brackets refer to the averaging of *N*, *C*, and *S* over the measurement area, which can cause depolarization (*D* > 0 and *P* < 1) if the sample is inhomogeneous. This happens when a MXene flake contains regions with different thicknesses (for example, from different numbers of Ti_3_C_2_T_*x*_ layers) within the SME spot. These thickness variations are described in the ellipsometric optical model by the thickness non-uniformity (NU).^59–61^

### Details on SME Data Analysis

The ellipsometry data (Ψ, Δ, *D*) of all measured MXene flakes were evaluated in WVASE v3.668 and CompleteEASE (CE) v6.75b (J.A. Woollam Co., Inc.). Leveraging the advantages of a multi-sample analysis,^61^ we identified flake-specific parameters (thickness *d*, non-uniformity NU, and resistivity ρ), while keeping the remaining model parameters identical for all flakes. The dielectric functions ϵ­(*E*) of the flakes were fitted as the sum of a Drude oscillator and two harmonic oscillators,

2 $$\epsilon \left(E\right)=\epsilon_{\infty }+\epsilon_{\mathrm{Drude}}\left(E\right)+\epsilon_{\mathrm{Harm}}^{\mathrm{NIR}}\left(E\right)+\epsilon_{\mathrm{Harm}}^{\mathrm{UV}}\left(E\right),$$

with the Drude contribution given by

3 $$\epsilon_{\mathrm{Drude}}\left(E\right)=-\frac{\hbar^{2}}{\varepsilon_{0}\rho \left(\tau E^{2}+i\hbar E\right)}$$

­(reduced Planck constant ℏ, vacuum permittivity *ε*_0_, resistivity ρ, mean scattering time τ, photon energy *E*), and the harmonic oscillators given by

4 $$\epsilon_{\mathrm{Harm}}^{j}\left(E\right)=\frac{A_{j}B_{j}E_{j}}{E_{j}^{2}-E^{2}-iB_{j}E}$$

­(amplitude *A*_*j*_, broadening *B*_*j*_, center energy *E*_*j*_). The UV oscillator serves a dual purpose. First, it describes absorption features (associated with interband transitions from mixed Ti_3_C_2_T_*x*_ terminations) that occur in the UV tail and outside of the SME-accessible spectral range. Second, it mimics the effect of the high-frequency dielectric constant ϵ_∞_, allowing us to set ϵ_∞_ = 1. This reduces the number of free fit parameters and minimizes parameter correlations.

Thickness non-uniformity was modeled as linear thickness variations within the SME spots (“rectangular profile” in CE), with five thickness points equally spaced around the mean flake thickness *d*. The fitted CE-internal percentage values NU_%_ were converted into thickness values NU_*d*_ via NU_*d*_ = 0.276·*d*·NU_%_. Non-uniformities in terms of NoL were calculated according to NU_NoL_ = NU_*d*_/*d*·NoL. The average numbers of Ti_3_C_2_T_*x*_ layers within the SME spots were derived from NoL­(*d*) = 1 + (*d* – *d*_mono_)/*d*_step_, based on the monolayer thickness *d*_mono_ = 1.40 nm and the step thickness *d*_step_ = 1.25 nm (for each additional layer) obtained from the MSA.

In summary, the common fit parameters shared by all the flakes were *A*_*j*_, *B*_*j*_, and *E*_*j*_ of the UV and NIR oscillator and τ of the Drude oscillator, whereas the flake-specific (*F*) parameters within the MSA were *d*(*F*), NU_%_(*F*) and ρ­(*F*). NoL­(*d*), NU_*d*_, NU_NoL_, and sheet resistance *R* = ρ/*d* are derived quantities.

### SME Error Analysis and Alternative Optical Models

Flake thicknesses *d* are fitted with high accuracy due to the sharp SiO_2_-related interference feature in the Ψ and Δ spectra (Figures 2 and S1), resulting in fit uncertainties below ±0.03 nm. Uncertainties for resistivities ρ are generally small for flakes with four layers or more, but increase for 3L, 2L, and 1L flakes. These uncertainties were upscaled by a factor of 5 (Figures 4 and S5) to reflect the potential coupling between ρ and τ. Because our MSA includes multiple ultrathin flakes with the same NoL, we could calculate the corresponding average resistivity values with reduced uncertainties. Non-uniformity values (NU_*d*_ and NU_NoL_) are also fitted with high confidence, as SME measures both Ψ/Δ and depolarization spectra *D* at multiple angles simultaneously. The NU uncertainties are highly flake dependent (Figure 6a) and reflect how well the linear thickness profile approximates the actual distribution of thicknesses within the SME spots.

To address a potential coupling between the fit parameters of the NIR and Drude oscillator (both situated at the edge of the probed spectral range), we tested a simplified alternative model without a Drude term, but with a flake-dependent amplitude of the NIR oscillator. We found that this model cannot adequately reproduce the ellipsometric spectra (especially in the NIR region), resulting in a larger MSE (reduced mean squared error), in an unphysically pronounced flake-to-flake scattering among oscillator-amplitude data points, in unreasonably high amplitude values (20% to 60% higher than expected from broadband measurements of Ti_3_C_2_T_*x*_ films^44^), and in an oscillator center energy redshifted from 1.50 eV (826 nm) to 1.41 eV (880 nm), inconsistent with recently reported literature values for macroscopic films.^11,21,39,41–44^ From these observations, we deduce that a Drude oscillator should be included in the optical model. This decoupling of the two oscillators can also be inferred from Figure 3, which shows that the spectral range of the SME is large enough to capture the inflection point of the NIR oscillator lineshape in ϵ_2_. The ellipsometric spectra are thus affected by both the NIR oscillator and the contribution of the Drude tail, enabling a sufficiently sensitive fit for both.

As previous studies of macroscopic Ti_3_C_2_T_*x*_ films indicate a potential thickness dependence of the NIR interband transition,^40,42,43^ we also tested a model with flake-dependent NIR oscillator amplitude and Drude resistivity. This model exhibits correlated fit parameters, but hints at a small increase in amplitude and an even more pronounced increase in resistivity toward monolayer flake thicknesses, consistent with our four-probe measurements.

Previous studies of macroscopic multi-flake films also indicate a dependence of the optical and transport properties on humidity.^59,62^ We performed SME measurements on Ti_3_C_2_T_*x*_ flakes at decreasing relative humidity using a continuous stream of nitrogen gas, but did not observe any significant changes in these properties, indicating that intercalated water cannot be easily removed from within the flakes.

We finally note that it was found not necessary to include in the model a low-refractive-index layer between the oxide and MXene, which could be used to account for inadequate flake adhesion. All investigated MXene flakes appear to have adhered very well to the substrate, as confirmed by the STEM measurements in Figures 5 and S7 where no partial detachment or complete separation from the oxide is observed within the SME spots.

### Complementary Nanoscale Methods

AFM measurements (Dimension Icon XR Scanning Probe Microscope, Bruker) were performed in tapping mode. An Sb-doped silicon tip (RTESP-75) with a spring constant of 3 N/m and a resonance frequency of 75 kHz was used for the measurements.^28,63^ AFM images were evaluated in Gwyddion 2.67. Images were corrected for linear tilts. Topography baselines were set to 0 nm (black) at the bare-substrate level. AFM thicknesses (Figure S2) were calculated as the average heights within the SME spots (indicated with circles in Figure S7). AFM images in Figures 5 and S7 were cropped around the flakes for clarity, and the height ranges were reduced for enhanced NoL contrast.

Electrode patterns for four-probe resistivity measurements were created by electron-beam lithography, followed by the deposition of 5 nm Cr and 100 nm Au using a thermal evaporator system (VST), and gentle lift-off in warm acetone. Current was injected through the two outer channels using a Keithley 2400 SourceMeter, while the voltage drop across the two inner probes was measured with a Keithley 2000 Multimeter.

A focused ion beam (FIB) (Helios Nanolab 460F1 Lite, Thermo Fisher Scientific)^57,64^ was used to cut 100 nm thin cross-section lamellas of selected MXene flakes. The lamellas were transferred onto TEM grids with a micromanipulator and measured using an STEM (Themis Z G3 aberration-corrected STEM, Thermo Fisher Scientific, operated at 300 kV) equipped with a high-angle annular dark-field (HAADF) detector. High-resolution STEM measurements (HRSTEM) were performed along the {112̅0} and {11̅00} zone axes of Ti_3_C_2_T_*x*_. STEM images were evaluated in Velox (Thermo Fisher Scientific).

## Supplementary Material



## Notes

Ψ­(45°)/Δ­(45°)/*D*(45°) overview spectra; flake thicknesses (SME, AFM, STEM); dielectric function and optical constants (eV); transmission and absorbance spectra; flake resistivity and sheet resistance; additional AFM and STEM images; HRSTEM images under different flake orientations (PDF)
