# Supplementary material for: Optical, Structural, and Charge Transport Properties of Individual Ti3C2T x MXene Flakes via Micro-Ellipsometry and Beyond
Source: ACS Nano. 2025 Sep 30;19(40):35414–24. doi: 10.1021/acsnano.5c06938 (PMC12530056; doi:10.1021/acsnano.5c06938)
Supplement: Supplementary file 1 [file nn5c06938_si_001.pdf]

Optical, Structural, and Charge Transport Properties of Individual $Ti_3C_2T_x$ MXene Flakes via Micro-Ellipsometry and Beyond

—

Supporting Information

Ralfy Kenaz,*[,1] Saptarshi Ghosh,[1] Mailis Lounasvuori,[2] Namrata Sharma,[2] Sergei Remennik,[3] Atzmon Vakahi,[3] Hadar Steinberg,[1,3] Tristan Petit,[2] Ronen Rapaport,[1] and Andreas Furchner*[,2]

[1] *Racah Institute of Physics, The Hebrew University of Jerusalem, Jerusalem 9190401, Israel*

[2] *Helmholtz-Zentrum Berlin für Materialien und Energie GmbH, Nanoscale Solid–Liquid Interfaces, Schwarzschildstraße 8, 12489 Berlin, Germany*

[3] *Center for Nanoscience and Nanotechnology, The Hebrew University of Jerusalem, Jerusalem 9190401, Israel*

* E-mail: ralfy.kenaz@mail.huji.ac.il; andreas.furchner@helmholtz-berlin.de

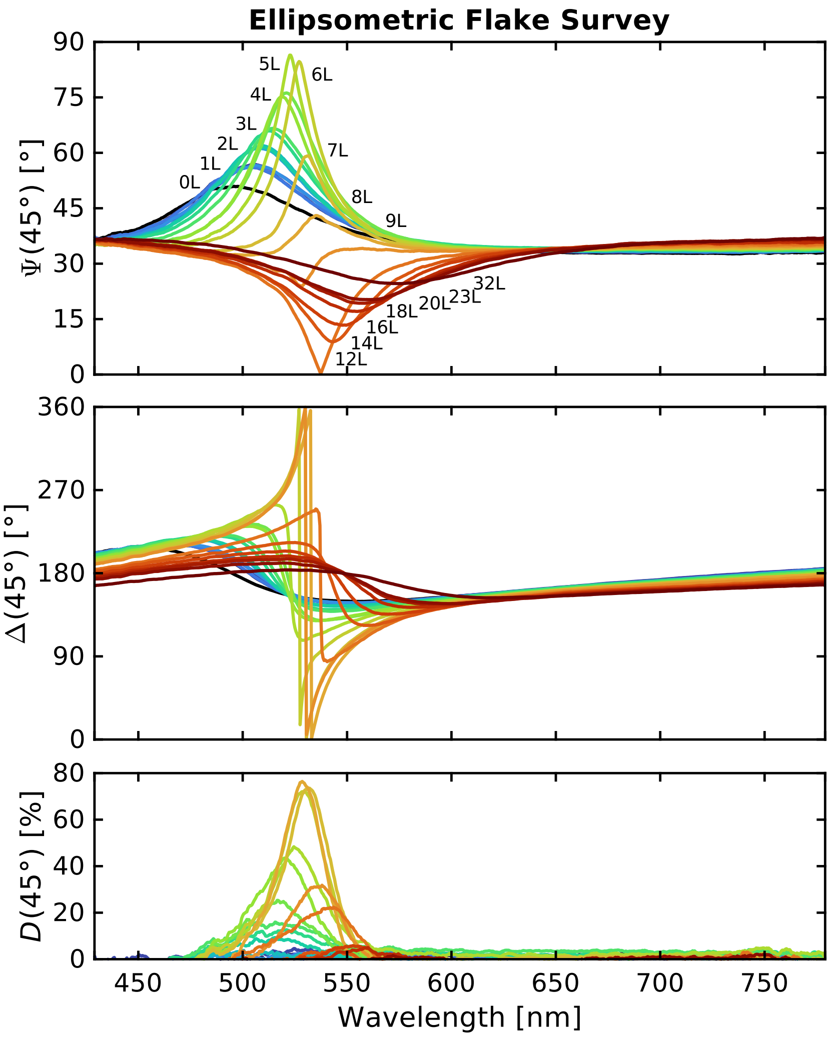

Figure S1: Ellipsometric Ψ, Δ, and D spectra of all 24 investigated $Ti_3C_2T_x$ MXene flakes, measured at 45° incidence angle.

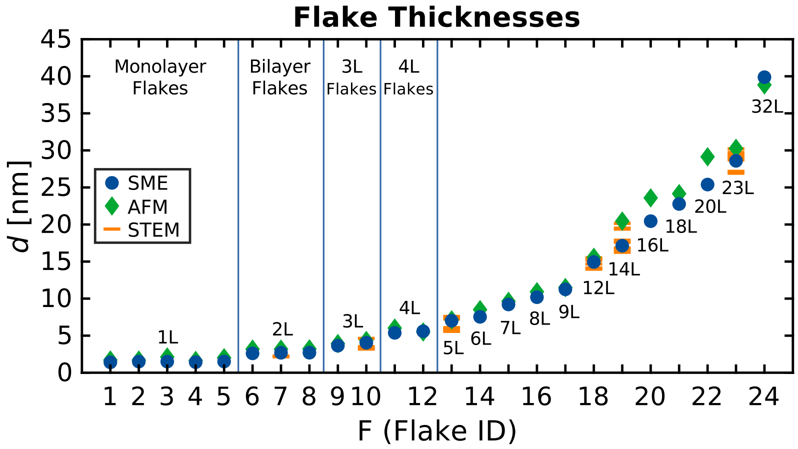

Figure S2: Thicknesses of all 24 flakes, as determined via SME, AFM and STEM (for selected flakes).

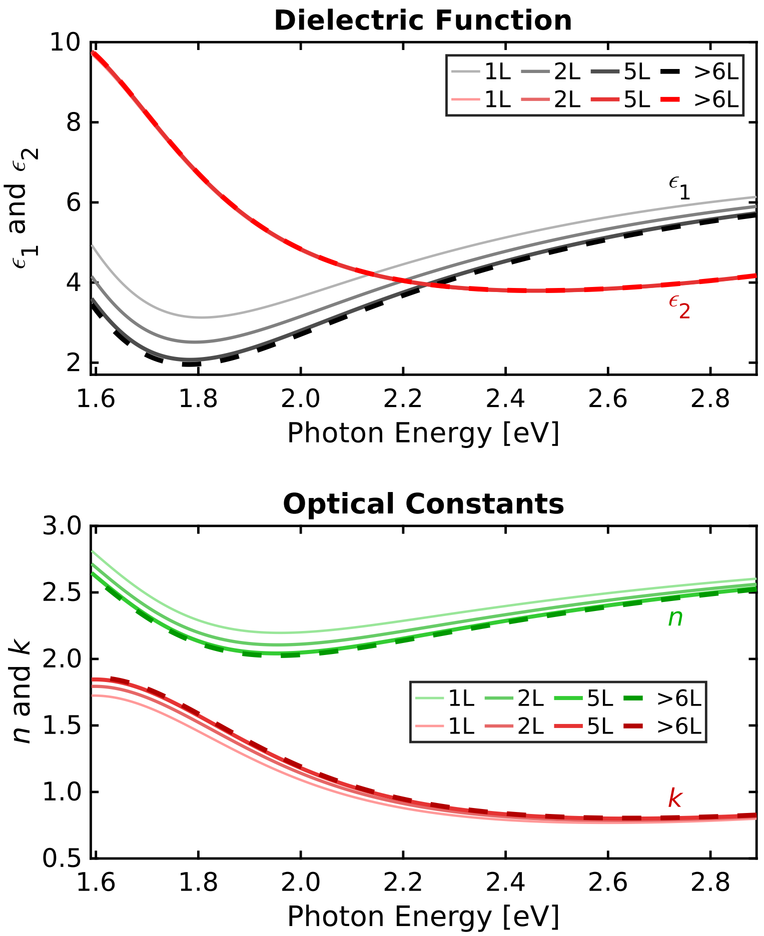

Figure S3: Dielectric function and optical constants (eV scale) for flakes with different number of layers.

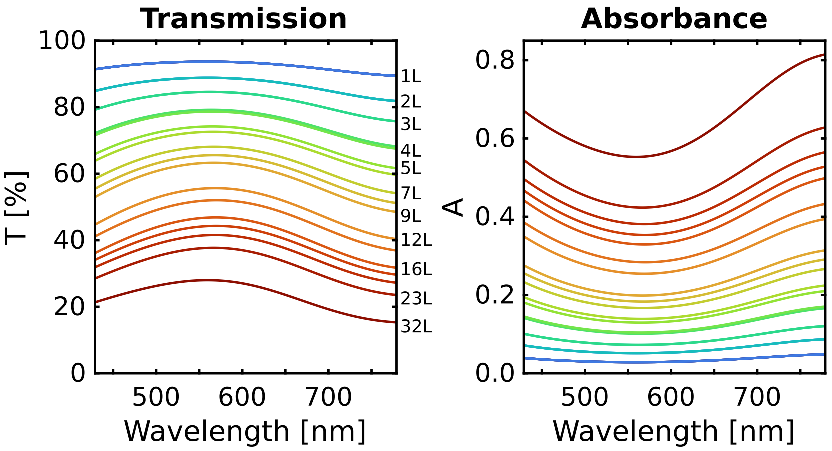

Figure S4: Calculated transmission and absorbance spectra of selected flakes (without substrate), based on the ellipsometric multi-sample analysis.

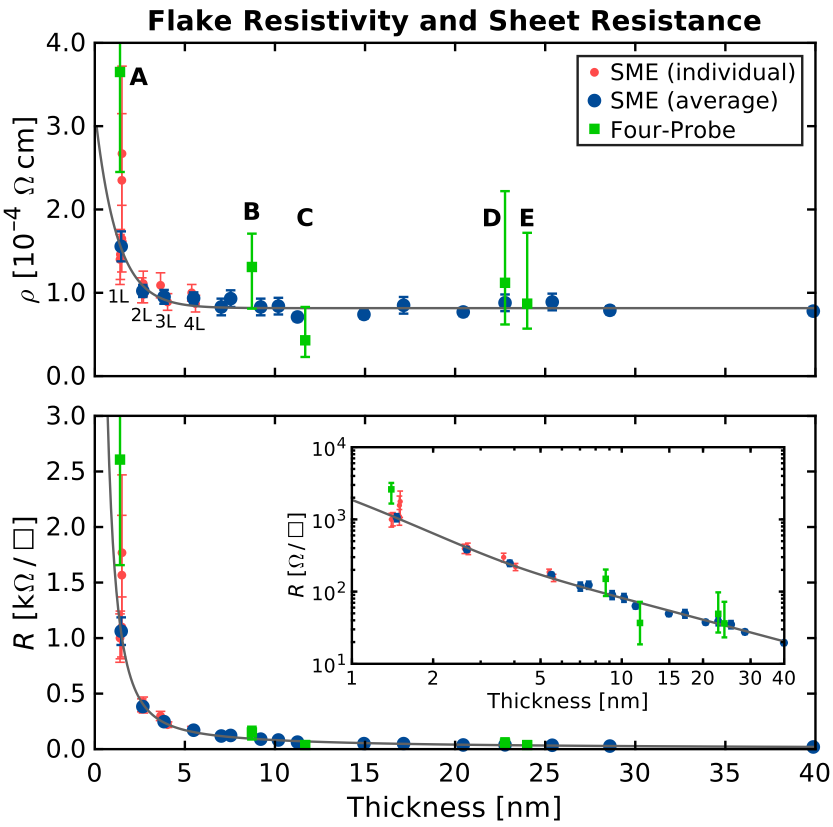

Figure S5: Thickness-dependent flake resistivity ρ and sheet resistance R (double log scale as inset) from ellipsometry and from direct electrical four-probe measurements (optical images of flakes A–E in Figure 4b). SME data of the 1L, 2L, 3L, and 4L flakes are shown both as averages and individually for each flake.

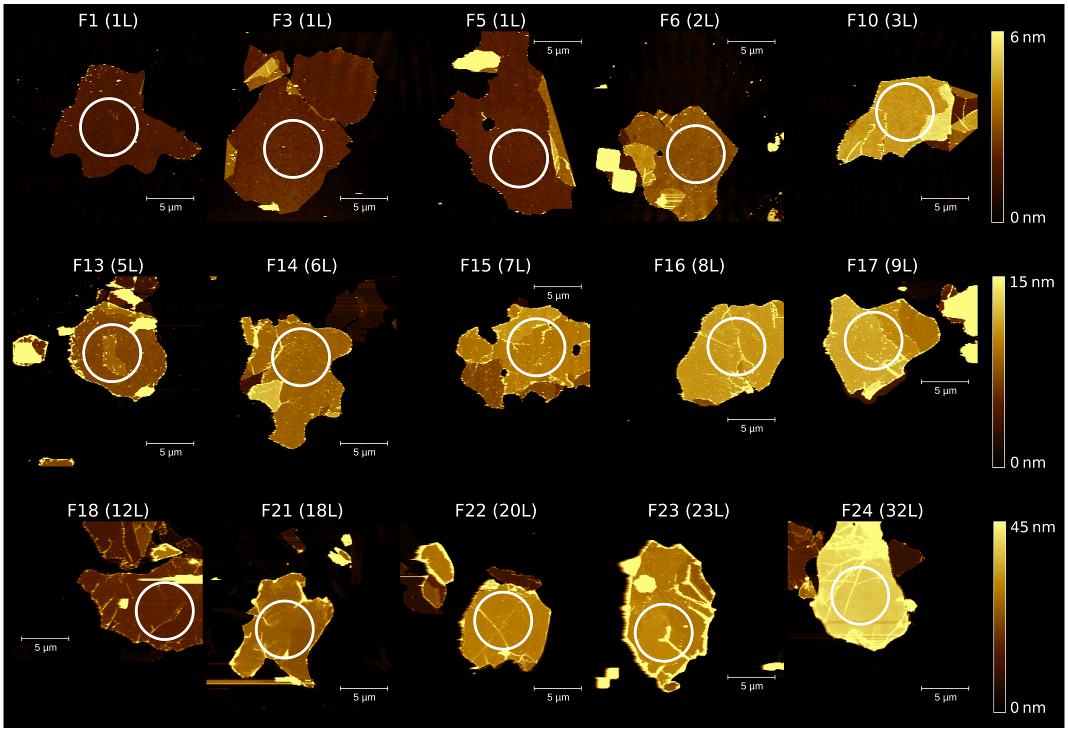

Figure S6: AFM images of representative flakes with Flake IDs (see Figure S2) and number of layers (NoL). SME measurement spots are marked with circles. A gold marker (two squares), used for easy flake cartography, is visible left to Flake 6.

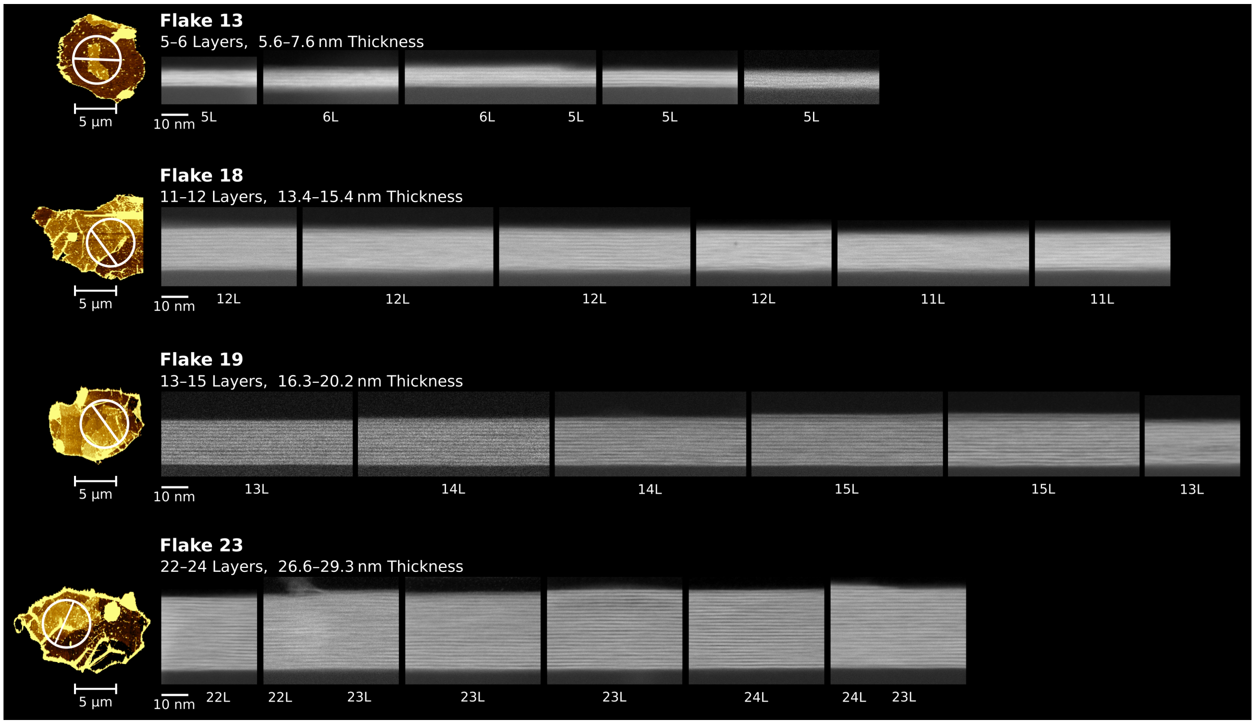

Figure S7: AFM topography and STEM HAADF cross-section images of flakes F13, F18, F19 and F23, taken from cuts (left to right along the marked lines) across the SME measurement spots (circles). This figure provides an extended set of STEM images beyond those shown in Figure 5.

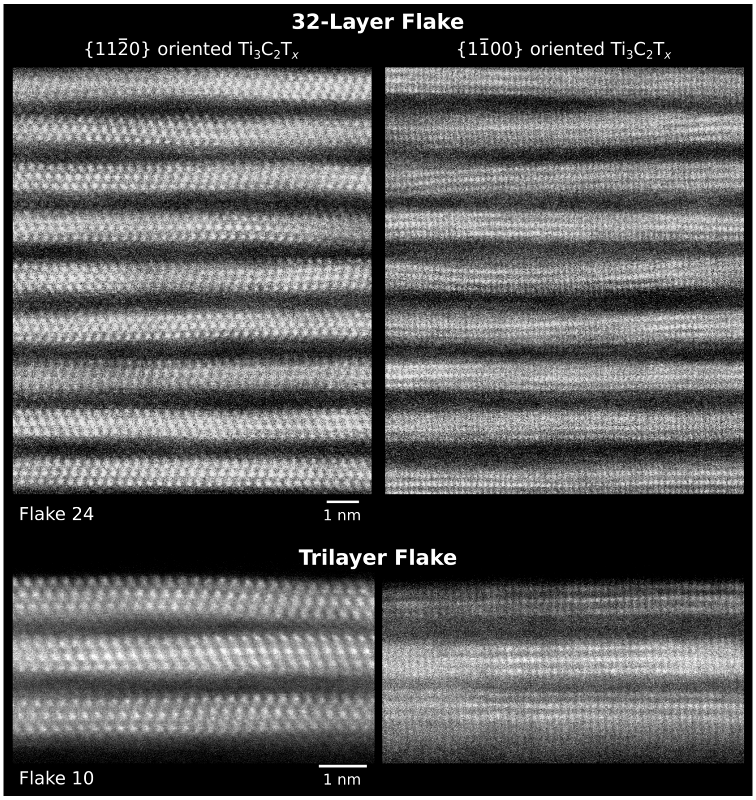

Figure S8: HRSTEM HAADF images of part of a 32-layer flake and of a trilayer flake in $\{11\bar{2}0\}$ and $\{1\bar{1}00\}$ orientation of $Ti_3C_2T_x$, showing the characteristic zigzag pattern and stacked pattern of Ti atoms, respectively (as expected from a $P6_3/mmc$ space-group material), as well as non-uniformity in the interlayer spacing.
